# The Shh Signaling Pathway Is Upregulated in Multiple Cell Types in Cortical Ischemia and Influences the Outcome of Stroke in an Animal Model

**DOI:** 10.1371/journal.pone.0124657

**Published:** 2015-04-30

**Authors:** Yongmin Jin, Nataly Raviv, Austin Barnett, Nicholas C. Bambakidis, Emily Filichia, Yu Luo

**Affiliations:** Department of Neurological Surgery, Case Western Reserve University, Cleveland, United States of America; University of South Florida, UNITED STATES

## Abstract

Recently the sonic hedgehog (shh) signaling pathway has been shown to play an important role in regulating repair and regenerative responses after brain injury, including ischemia. However, the precise cellular components that express and upregulate the shh gene and the cellular components that respond to shh signaling remain to be identified. In this study, using a distal MCA occlusion model, our data show that the shh signal is upregulated both at the cortical area near the injury site and in the adjacent striatum. Multiple cell types upregulate shh signaling in ischemic brain, including neurons, reactive astrocytes and nestin-expressing cells. The shh signaling pathway genes are also expressed in the neural stem cells (NSCs) niche in the subventricular zone (SVZ). Conditional deletion of the shh gene in nestin-expressing cells both at the SVZ niche and at the ischemic site lead to significantly more severe behavioral deficits in these shh iKO mice after cortical stroke, measured using an automated open field locomotion apparatus (Student’s t-test, p<0.05). In contrast, animals given post-stroke treatment with the shh signaling agonist (SAG) demonstrated less deficits in behavioral function, compared to vehicle-treated mice. At 7 days after stroke, SAG-treated mice showed higher values in multiple horizontal movement parameters compared to vehicle treated mice (Student’s t-test, p<0.05) whereas there were no differences in pre-stroke measurements, (Student’s t-test, p>0.05). In summary, our data demonstrate that shh signaling plays critical and ongoing roles in response to ischemic injury and modulation of shh signaling in vivo alters the functional outcome after cortical ischemic injury.

## Introduction

Stroke is one of the leading causes of death and disability worldwide and places a heavy burden on the economy in our society. Currently, the only FDA-approved treatment for stroke is thrombolytic therapy with tissue-type plasminogen activator (tPA) which is only effective when delivered in a narrow time window after stroke onset. [1, 2]. Both neurogenesis from the subventricular zone (SVZ) niche and in situ activated astrocytes that have stem cell properties have been implicated in the repair process after brain injury including ischemia [3, 4]. Understanding the mechanisms that control the repair process, involving both the endogenous stem or progenitor cells both at the SVZ and at the injury sites may provide insights into therapeutic interventions that modulate intrinsic process of neuroregeneration and lead to potential enhanced repair and functional recovery.

As a key modulator in the maintenance of neuronal stem cells, sonic hedgehog (shh) signaling plays important roles in the self-renewal, proliferation, and migration of these cells [5–11]. Shh signaling also controls the generation and survival of oligodendrocytes [12, 13]. Recent studies have also highlighted the critical role for shh in modulating blood brain barrier (BBB) integrity [14, 15] and reactive astrocytes in response to brain injury [16, 17]. We and others have shown the beneficial effects of shh activation in brain injury animal models, including stroke [18–21]. Shh signaling is activated in response to central nervous system (CNS) injury; however, the precise cell types that upregulate shh secretion and the cellular components that respond to upregulated shh signaling remain to be elucidated. In this study, we characterized the cellular components that participate in cortical ischemia- induced shh signaling activation. Utilizing an inducible conditional knockout mouse model, we examined the functional outcome in cortical stroke after conditional deletion of shh genes in nestin-expressing cells both at the SVZ and injury sites. A shh pathway agonist (SAG) has previously been shown to activate shh signaling and control the proliferation of progenitor cells in vivo [22, 23]. In this study we also examined the effects of shh signaling activation on the functional outcome after stroke using this agonist.

## Materials and Methods

### Animals

All animal protocols were conducted under National Institutes Health (NIH) Guidelines using the NIH handbook *Animals in Research* and were approved by the Institutional Animal Care and Use Committee (Case Western Reserve University). All surgery was performed under isoflurane anesthesia, and all efforts were made to minimize suffering including the usage of slow release buprenorphine as postoperative analgesia and proper euthanization of animals following the NIH guidelines. The mice were housed in the animal facility of Case Western Reserve University on a 12-h light/dark diurnal cycle. Food was provided ad libitum. A pair of breeding colony of Nestin-CreERT2 mice were kindly provided by Dr. Amelia Eisch (University of Texas Southwestern Medical Center) and R26R-stop-YFP mice, a Cre recombinase reporter strain, were purchased from Jackson Laboratory. The generation and characterization of the nestin-CreERT2-R26R-YFP strain used in this study have been previously described [24]. Floxed shh mice [25] (B6;129-*Shh* ^*tm2Amc*^/J) mice were purchased from Jackson Laboratory. Nestin-creERT2 mice were crossed with floxed shh mice to generate viable and developmentally normal adult nestin-CreERT2/shh wildtype (wt) (nestin-CreERT2+/ Shh^wt/wt^) or shh inducible knockout (iKO) mice (nestin-CreERT2+/ Shh^loxP/loxP^). Mice were genotyped for nestin-CreERT2, R26R-stop-YFP allele and shh floxed allele by primers described in the Jackson Laboratory colony information.

### Tamoxifen administration

Male nestin-CreERT2-R26R-YFP mice (6–8 weeks old) were given tamoxifen (TAM) dissolved in 10% EtOH/90% sunflower oil by gavage feeding at a dose of 180 mg/kg daily for 5 consecutive days. Control mice received vehicle. This dosing regimen has been previously demonstrated to provide maximal recombination with minimal mortality [24]. For neural stem cell (NSCs) fate mapping, TAM treated mice received distal Middle cerebral artery occlusion (MCAo) surgery 2 weeks after the last TAM administration. Aged Shh iKO mice and their wildtype (wt) littermates (20-22months old male) received the same treatment regimen of tamoxifen except the time before distal MCAo was only 3 days.

### Cortical ischemia model (distal MCA occlusion)

Focal cerebral ischemia was produced in the mice using the procedure we described [26, 27]. The mice were anesthetized with isoflurane. Body temperature was monitored and maintained at 37· C by a heating pad. The surgical area was shaved and prepared with alternating betadine scrubs and ethanol. A small 5-mm vertical skin incision was cut between the right eye and ear to expose the skull. A craniotomy of about 1 × 1 mm^2^ was made in the right squamosal bone to expose the middle cerebral artery (MCA). The MCA was lligated with 10–0 suture for 90min followed by removal of the ligating suture to allow for reperfusion. The skin wound was closed with sutures and after recovery from the anesthesia, body temperature was maintained at 37°C using a temperature-controlled incubator.

To compare the migration of SVZ derived neuroblasts into the ischemic area after distal and proximal MCAo, a small group of mice (n = 3) were subjected to a proximal MCA occlusion using an intraluminal filament as previously described [28].

### Behavioral assay

Animals were placed in an Accuscan activity monitor (Columbus, OH) before and 7 days after MCAo for behavioral recording for 24hours. The monitor contained 16 horizontal and 8 vertical infrared sensors spaced 2.5 cm apart. Each animal was placed in a 42×42×31 cm plexiglass open box for 60min or 24hours with food and water available. Motor activity was calculated using automated Versamax software (Accuscan). The following variables were measured: (A) horizontal activity (the total number of beam interruptions that occurred in the horizontal sensors), (B) total distance traveled (the distance, in centimeters, traveled by the animals) and (C) total movement number (the total number discrete horizontal movements).

### Triphenyltetrazolium chloride (TTC) staining

Two days after MCA ligation, some animals were sacrificed and perfused intracardially with saline. The brain tissue was then removed, immersed in cold saline for 5 minutes, and sliced into 1.0 mm thick sections. The brain slices were incubated in 2% triphenyltetrazolium chloride (TTC, Sigma), dissolved in normal saline, for 10 minutes at room temperature, and then transferred into a 5% formaldehyde solution for fixation.

### Experimental timeline (Fig 1A)

For shh signaling pathway gene expression visualization in different cell types after ischemic injury, young adult male (6–8 weeeks old) NestincreERT2- R26R-YFP mice were given TAM for 5 days and 14 days later distal MCA occlusion was carried out. At 14 days post stroke, we examined the expression of shh signaling pathway gene expression in the cortex, striatum and SVZ area both contralateral and ipsilateral to the ischemic side. To compare the migration of newly generated cells from SVZ into the ischemic area in cortical and striatal stroke models, two groups of mice that received either distal MCAo or intraluminal filament MCAo were studied after survival for 30 days.

A cohort of aged (20–22 month old) male shh conditional knockout mice, nestin-CreERT2(+)/shh^loxP/loxP^ (n = 8) and wildtype mice, nestin-CreERT2(+)/ Shh^wt/wt^ (n = 7, one mouse died during the process of aging), were subject to 5-day TAM treatment 3 days before the stroke surgery. Investigators were blinded as to the genotype of the mice when surgery was performed. All mice (n = 8 for shh iKO and n = 7 for wt mice) survived TAM treatment and MCAo surgery. Behavioral measurements were carried out 2 days before stroke surgery and 7 days after stroke surgery.

For shh agonist treatment, young adult (10 weeks old) C57bl6/J male mice were subjected to MCA occlusion. Behavioral measurements were carried out before stroke surgery to record the baseline locomotor function in all mice. On day3 post stroke, all the mice were subjected to a brief 1 hour open field behavioral evaluation and were randomly grouped into vehicle or smo agonist (SAG) treatment groups (n = 15 for each group). After randomization, the pre-stroke behavioral scores and the 1hour post-stroke day (psd 3) scores were calculated to ensure that there was no significant difference in pre-stroke and psd3 behavioral measurements between the two groups. Starting from post-stroke day 3, mice received either smo agonist [22] treatment (10mg/kg/day) or vehicle (n = 15 for each group) until psd7. Behavioral measurements were carried out again at 7 days post stroke. Investigators who placed the mice into the behavioral open field chambers were blinded to the genotype and the treatment of the mice. The behavioral tests we used in this study are completely controlled by the computer operated VersaMax program after the mice were placed into the open field chambers to avoid potential bias or interference by observers.

### Immunohistochemistry

2, 14 and 30 days after MCA occlusion, mice were perfused transcardially with a solution of 4% paraformaldehyde (PFA, pH 7.2) in 0.1 M phosphate buffer (PB, pH 7.2). Brains were removed from the skull, postfixed in 4% PFA overnight at 4°C, and were processed with ethanol (70%, 95% and 100%) and xylene prior to paraffin embedding. After paraffin embedding, 5-μm sections were mounted on slides to be used for immunohistochemical analyses. Paraffin-embedded sections were deparaffinized with xylene and rehydrated with ethanol before undergoing antigen retrieval. The sections were then incubated with blocking buffer for one hour. The primary antibodies were prepared in the blocking buffer and the sections were incubated in the solution overnight. The antibodies used were chicken monoclonal anti-Nestin (1:1000; R&D), rabbit monoclonal anti-GFAP (Glial fibrillary acidic protein) (1:500; Invitrogen), mouse monoclonal anti-MAP-2 (1:500; Millipore), affinity-purified goat anti-Dcx (Doublecortin) (1:100; Santa Cruz Biotechnology), mouse monoclonal anti-NeuN (1:500; Millipore), and mouse monoclonal anti-PCNA (Proliferating cell nuclear antigen) (1:400; Dako). As the endogenous YFP fluorescence does not survive fixation and paraffin embedding processes, for YFP detection, we used a rabbit anti-GFP antibody that also recognizes YFP protein (1:3000, Invitrogen). A goat polyclonal anti-SHH antibody (N-19) raised against the N-terminus of human SHH precursor (1:50), a goat polyclonal anti-PTCH (C-20, raised against the C-terminal 20 amino acids of the human protein; 1:200), and a rabbit polyclonal anti-SMO (H-300, raised against amino acid residues 488–787 of the human protein; 1:100) were all purchased from Santa Cruz (Santa Cruz, CA, USA). These antibodies have been used previously and specificity has been confirmed by Western blotting [29–32]. In particular, the anti-SHH antibody is specific for the 52 kDa SHH precursor protein, rather than the active, secreted morphogen (19 kDa), and only labels cells that are the source of morphogen. After incubation with primary antibody solution, the sections were washed and incubated for one hour at room temperature in diluted secondary antibody prepared with blocking solution [donkey anti-rabbit 488, anti-goat 555, anti-mouse 488 (1:1000/500; Life Technologies); donkey anti-chicken FITC (1:1000; Fisher Scientific)]. The slides were then washed with 0.1% Triton-X100 in TBS (tris buffered saline) and coverslipped. Images were acquired using an Olympus microscope. Omission of the primary or secondary antibodies resulted in no staining and served as negative controls. Shh, GFAP and nestin immunoreactivity in the cortical ischemic area and adjacent striatal area were quantified using Nikon NIS-Elements software and averaged from 3 sections for each animal on both contralateral and ipsilateral stroke sides of the brain. Colocalization of shh, GFAP and nestin fluorescent immunostaining in the cortical ischemic area was analyzed using the colocalization function in Nikon NIS-Elements software and presented as a scatterplots of the two signals, analyzed with Pearson’s correlation coefficient (PCC) as a statistic for quantifying colocalization. PCC values ranged from -1 to 1 with PCC = 0 reflecting distributions of two signals that are uncorrelated with one another and 1 reflecting colocalization perfectly linearly related to each other. Because striatal shh expressing cells are scattered and easily distinguishable as single cells, colocalization of shh, GFAP and nestin expressing cells in striatal areas were counted in overlaying images using the cell counting function in Nikon NIS-Elements software and are presented as percentage of GFAP+/shh+ or nestin+/shh+ cells in the population of shh expressing cells. All immunohistochemical measurements were done by blinded observers.

### Statistics

Statistical analysis was performed using Student’s *t* test, *p* values less than 0.05 were considered significant. Data are presented as mean ± s.e.m.

## Results

### Distal MCAo results in a focal cortical stroke that induces minimal migration of newly generated neuroblasts into the injury site

**Fig 1:**
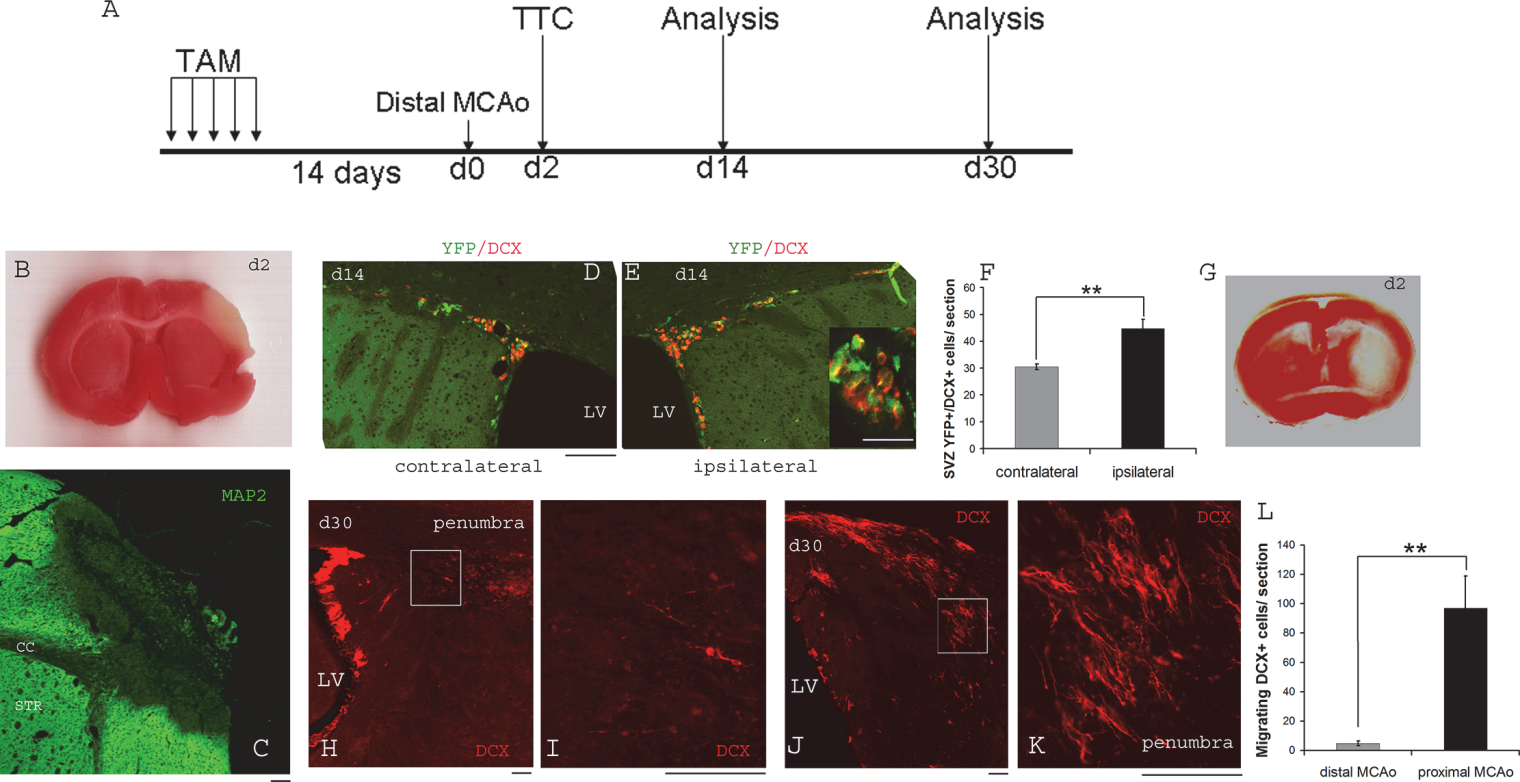
Expression of yellow fluorescent protein (YFP) and double cortin (DCX) in neural progenitor/neuroblast cells in stroke brain. A) experimental timeline. B) TTC staining at 48 hours after distal MCAo showing infarction restricted to cortical area. C) MAP2 immunostaining reveals loss of neurons in cortical stroke D) and E) YFP and DCX immunostaining in SVZ in the contralateral and ipsilateral sides of the brain. Inset shows the staining at higher magnification.. F) quantification of SVZ YFP+/DCX+ cells in the contralateral and ipsilateral side of the brain in distal MCAo mice (n = 6). G) TTC staining showing a representative image of infarction in proximal MCAo H) post-stroke day (psd 30) DCX immunostaining showing minimal migration of DCX+ positive cells into the ischemic site in distal MCAo. Boxed area in (H) is shown in panel I) at higher magnification. J) shows many migrating DCX+ neuroblasts toward the ischemic injury site in proximal MCAo. Boxed area in (J) is shown in panel K) at higher magnification. L) Quantification of total DCX positive cells per section at the ischemic sites in distal MCAo and proximal MCAo mice (n = 3–4 per group). Scale bar = 100um. ** indicates p<0.01, Student’s t-test.

We utilized a previously established protocol to fate map SVZ nestin-expressing NSCs in nestin-Cre ERT2/R26R-YFP mice. Li et al (2010) [33] investigated the mutilineage cytogenic response in a proximal MCAo stroke model which generates ischemic injury that is mainly restricted to striatum. In contrast, in this study we first examined the cytogenic response of SVZ NSCs to cortical ischemia using distal MCAo. TTC staining at 48hours post stroke revealed a focal infarction that is restricted to the cortical region (Fig 1B). MAP2 immunostaining confirmed the loss of neuronal cells and their projections in the infarcted area (Fig 1C). Similar to previous reports, this TAM treatment protocol specifically labeled nestin-expressing SVZ NSC cells with YFP protein. Yellow fluorescent protein (YFP+) and doublecortin (DCX+) cells at 14 days post cortical stroke showed increased numbers in the ipsilateral SVZ (Fig 1E) compared to contralateral SVZ (Fig 1D) (quantification of YFP+/DCX+ cells/section shown in Fig 1F, contralateral side = 30.5±1.09, ipsilateral side = 44.83±3.36, p = 0.002 Student’s t-test), showing that cortical stroke is able to stimulate the proliferation of SVZ NSCs. However, at post stroke days 30, there are only very few visible YFP+ or DCX+ cells in the cortical ischemic region (Fig 1H, 1I and 1L, number of DCX+ cells in the cortical ischemic area = 4.84±1.58/section). To compare the induction and migration of DCX+ neuroblasts in a striatal stroke, we also carried out proximal MCAo (Fig 1G) in nestin-Cre ERT2/R26R-YFP animals and compared DCX staining to that in the cortical stroke brain. In contrast to cortical stroke, proximal MCAo (striatal stroke) mice showed many DCX+ cells in the striatal ischemic site at 30 days after stroke (Fig 1J–1L, number of DCX+ cells at striatal ischemic area = 96.88±21.92/section, significantly higher than in the distal MCAo model, p = 0.005 Student’s t-test).

### Cortical stroke stimulates shh upregulation in both cortex and striatum

**Fig 2:**
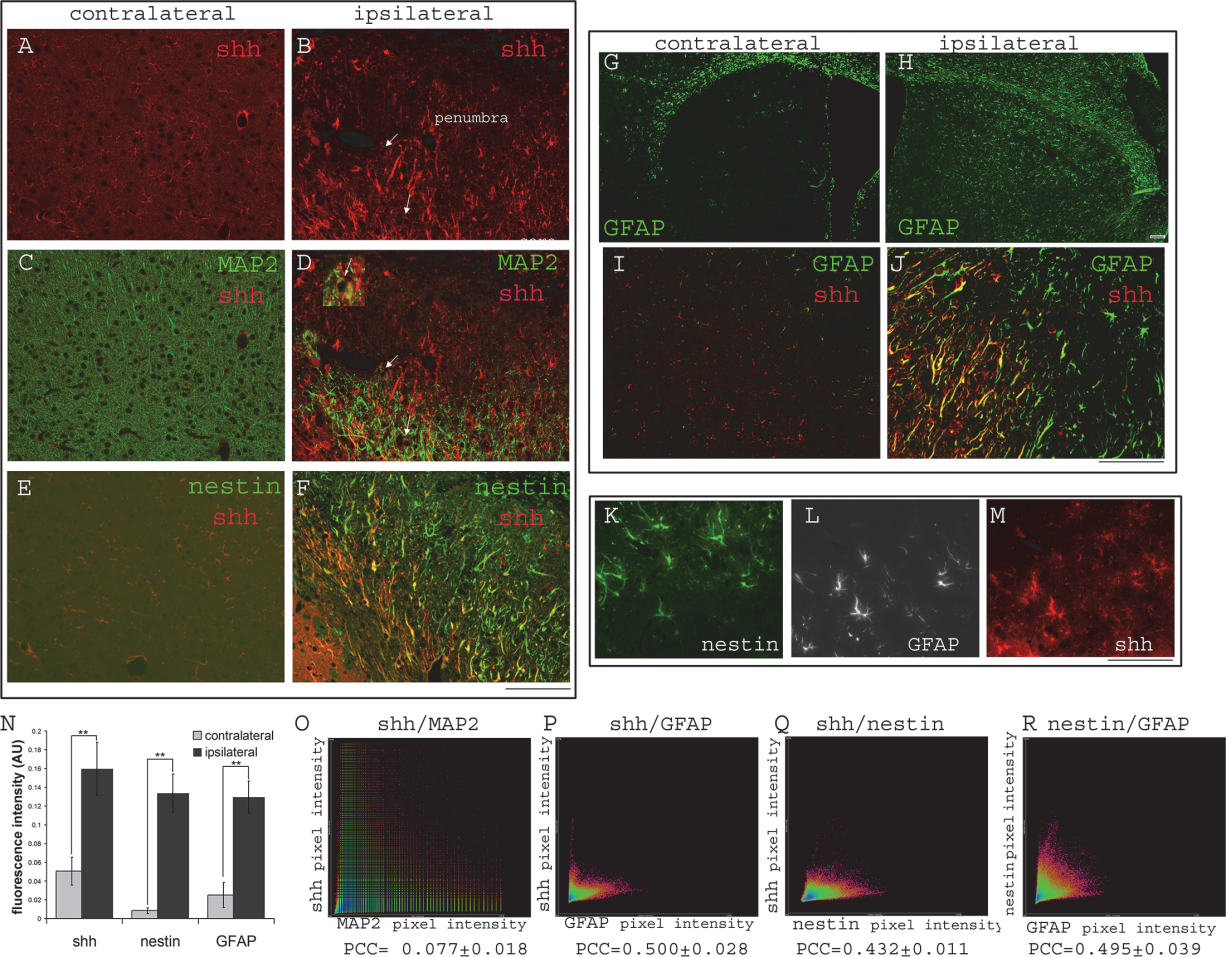
Distal MCAo induces shh expression in the cortical ischemic site. A) and C) expression of shh and MAP2 in the contralateral side cortex. B) and D) shh expression in the ischemic site of the ipsilateral cortex. Arrow and inset showing a few shh+/MAP2+ cells. E) basal expression of shh and minimal or no expression of nestin in contralateral side of cortex and F) shh and nestin upregulation near the ischemic site in ipsilateral cortex. G and H) upregulation of GFAP in both cortex and striatum in the ipsilateral side. I and J) expression of shh and GFAP in the contralateral and ipsilateral sides of cortex at higher magnification. K/L/M) staining of nestin/GFAP/shh on the same section. N) Quantification of immunofluorescent intensity for shh, nestin and GFAP in contralateral and ipsilateral sides of the cortical area (n = 6). O, P, Q and R) colocalization analysis of shh/MAP2, shh/GFAP, shh/nestin and nesin/GFAP signals (protein on the y axis to the protein on x axis as labeled on the scatterplots. Pearson’s correlation coefficient (PCC) are marked on the bottom of the scatterplots. Scale bar = 100um. ** indicates p<0.01, Student’s t-test. Mouse brains were analyzed at 14 days after distal MCAo.

We next examined shh expression in the cortical ischemic region. Contralateral cortex showed sparse shh+ cells that were not neurons (MAP2 negative) (Fig 2A–2C). In contrast, ischemic cortex demonstrated substantial upregulation of shh in many cells (Fig 2B, quantification of shh immunoreactivity in contralateral and ipsilateral cortical areas shown in Fig 2N). A small percentage (less than 10% of shh positive cells, total of >100 shh+ cells counted) of these shh expressing cells in the cortical stroke region were MAP2 positive neurons in the ischemic area (Fig 2D and inset). GFAP staining revealed that although the ischemic injury is restricted to the cortex, reactive GFAP+ astrocytes were present throughout both cortical and striatal regions in the stroke side (Fig 2G–2H). Double labeling with shh and GFAP protein revealed many shh+/GFAP+ cells in the cortical region near the ischemic site (Fig 2I contralateral side and Fig 2J ipsilateral side). In addition, we also observed strong nestin expression in the cortical ischemic site (Fig 2F) in cells that were also GFAP positive, consistent with the recent reports [4, 17] that reactive astrocytes might adopt stem cell properties after brain injury (Fig 2K–2M). Interestingly, many of the cells that upregulate nestin expression near the ischemic region were also positive for GFAP and shh expression (Fig 2F and 2K–2M), suggesting that shh signaling may be critical for these reactive astrocytes that respond to ischemic injury and upregulate both GFAP and nestin expression. Because shh protein expression is strongly upregulated in cortical ischemic sites and its immunostaining pattern makes it difficult to distinguish individual cell bodies, immunofluorescence intensity of shh, GFAP and nestin were measured in contralateral and ipsilateral cortical areas. Results show that all three proteins are significantly upregulated in the ipsilateral ischemic cortical area compared to the nonstroke side (Fig 1N, p<0.01 for all three proteins, Student’s t-test). Colocalization analysis demonstrated that shh staining mostly overlaps with GFAP staining (Fig 1P, Pearson’s correlation coefficient (PCC) = 0.500±0.028), as well as with nestin staining (Fig 1Q, PCC = 0.432±0.011) but correlates minimally with MAP2 staining (Fig 1O, PCC = 0.077±0.018), consistent with imaging observations. Interestingly, colocalization analysis of nestin and GFAP fluorescence signals showed a relatively high degree of overlap between these two proteins as well (Fig 1R, PPC = 0.495±0.035). It is important to note that since PCC quantifies the degree to which the variability in red and green pixel intensities co-occur proportionally. For signals that do not necessarily co-occur in fixed proportion to one another (for example shh expressing cells that also express GFAP do not necessarily always express these two proteins proportionally), PCC tends to yield lower values, often under-representing the degree of correlation between the two signals.

**Fig 3:**
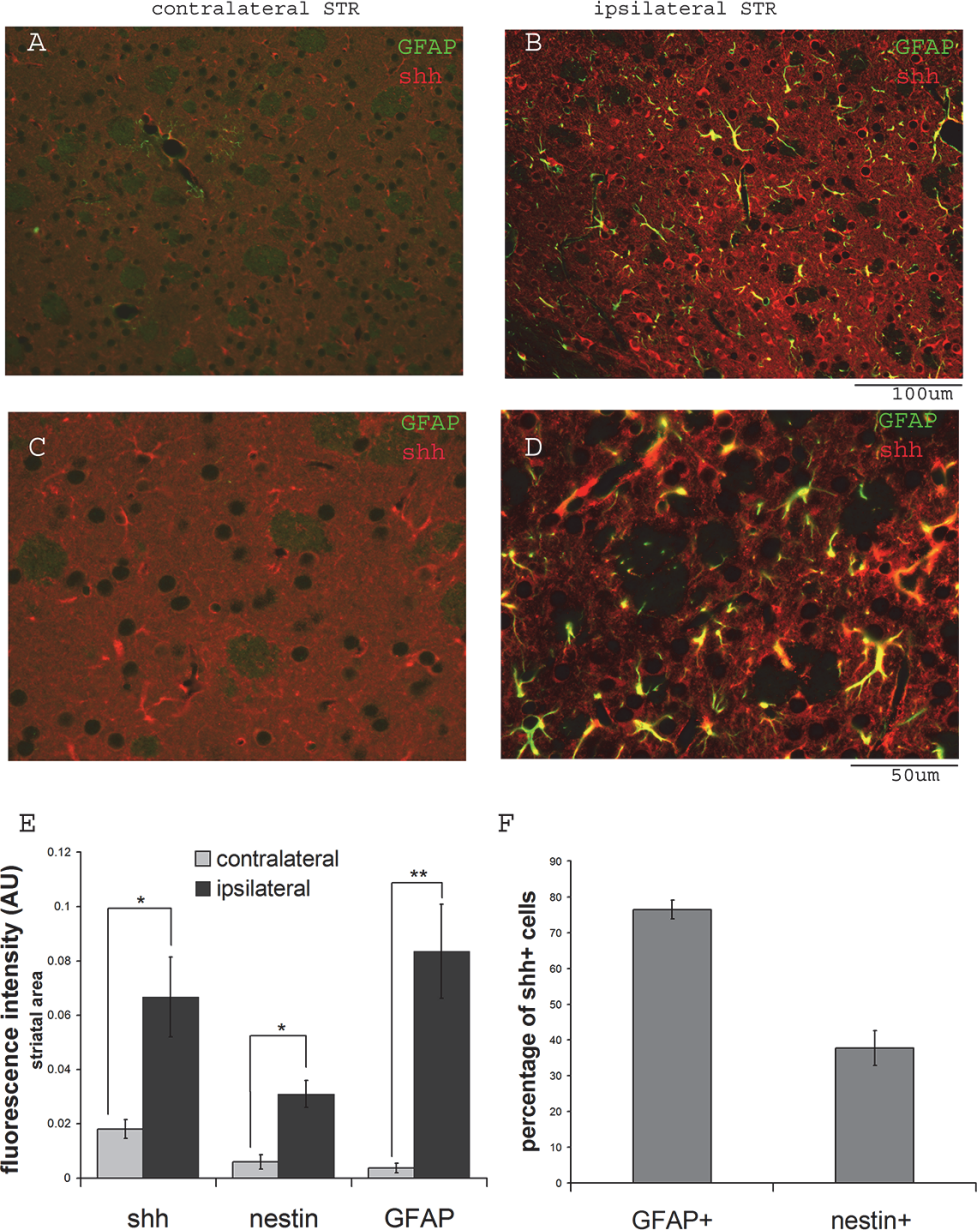
Upregulation of shh expression in the ipsilateral striatum in GFAP positive cells. A) Basal expression of shh in the contralateral striatum in GFAP negative cells, C) showing higher magnification. Shh and GFAP are upregulated in striatum in cortical distal MCAo model B). Higher magnification of shh+/GFAP+ cells are shown in panel D). E) Quantification of immunofluorescent intensity for shh and GFAP in contralateral and ipsilateral sides of the adjacent striatal area (n = 6). F) percentage of shh+ cells that are GFAP+ or nestin + in ipsilateral striatal area (>100 total cells counted). Scale bar is as marked. * indicates p<0.05 and ** indicates p<0.01, Student’s t-test. Mouse brains were analyzed at 14 days after distal MCAo.

**Fig 4:**
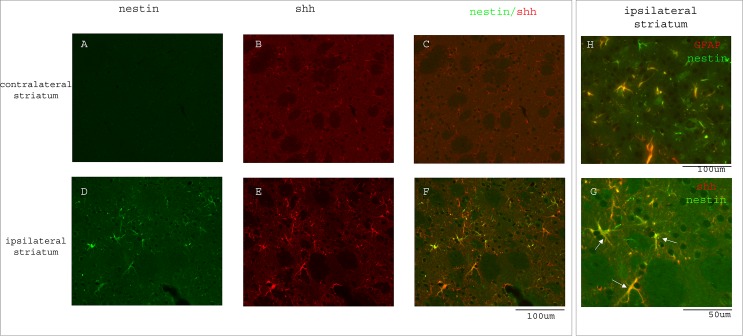
Nestin expression is induced in ipsilateral striatum. A.B.C) basal expression of shh and minimal or no expression of nestin protein in the contralateral striatum. D) nestin expression is upregulated in the ipsilateral striatum and many of the nestin+ cells are also shh+ (E and F). Higher magnification image is shown in panel G. (H) Some of the nestin-expressing cells in striatum are also GFAP positive. Scale bar is as marked. Mouse brains were analyzed at 14 days after distal MCAo.

We next examined shh expression in the striatal region on the contralateral and ipsilateral sides after ischemia. Although in the distal MCAo model, ischemic injury is only in the cortical region, shh, GFAP and nestin expression are significantly induced in the adjacent striatal region (Fig 3B–3D) compared to the contralateral side (Fig 3A–3C). Fig 4 shows representative images of nestin expression. Fig 3E shows the quantification of immunoreactivity intensity for contralateral and ipsilateral striatal areas for shh, GFAP and nestin expression, demonstrating higher expression levels in stroke side striatal areas for all three proteins (p = 0.018 for shh, p = 0.004 for GFAP and p = 0.011 for nestin, Student’s t-test). Because shh expressing cells in striatal area are scattered and easily distinguishable as single cells, we counted the percentage of GFAP+ or nestin+ populations in total shh+ cells. The majority of the shh expressing cells in striatum were GFAP+ reactive astrocytes (Fig 3D and 3F, 76.46±2.61% of total shh+ cells). Similarly, some shh+ cells were also nestin+ in the ipsilateral striatal area (Figs 3F and 4D, 4E and 4F, 37.72±4.89% of total shh+ cells). To examine whether these striatal nestin+ cells were reactive astrocytes, we also carried out double fluorescence immunostaining for GFAP and nestin proteins, and found that many of the reactive astrocytes in the ipsilateral striatum were nestin positive but some nestin+ cells were not GFAP+, suggesting nestin upregulation is not only restricted to GFAP+ reactive astrocytes (Fig 4H).

### The shh signaling pathway genes are expressed in the SVZ NSCs population

**Fig 5:**
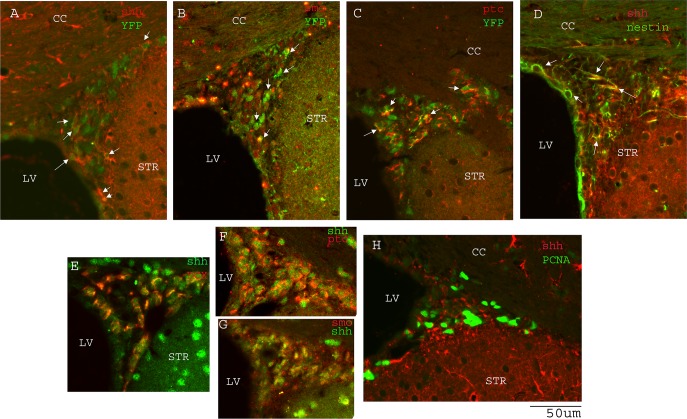
Shh signaling pathway genes are expressed in SVZ NSCs. A.B.C) shh, smo, ptc expression in YFP (+) cells in the SVZ. D) shh expression in nestin(+) NSCs in SVZ. E). shh expression is detected in DCX+ neuroblasts in SVZ. H) shh expression in PCNA(+) cells. F.G) coexpression of shh with smo, and shh with ptc in SVZ cells. Scale bar = 50um. Mouse brains were analyzed at 14 days after distal MCAo.

The shh signaling pathway has been implicated in the regulation and maintenance of the stem cell niche in SVZ. We next examined the expression of shh, its receptor patched1(ptc), and its effector smoothened (smo) expression in the SVZ NSCs niche. 14 days after TAM administration. In nestincreERT2-R26R-YFP mice, many YFP+ SVZ cells express shh protein both in the ipsilateral and contralateral sides of the brain (Fig 5A). Many YFP+ cells were also positive for smo (Fig 5B) and ptc (Fig 5C) immunostaining, suggesting that both the shh peptide and its receptor/effector genes are expressed in the SVZ niche. Further characterization of the cell types in the shh expressing cells in the SVZ niche showed that shh+ cells are positive for PCNA (proliferating cell nuclear antigen) immunostaining (Fig 5H), suggesting that shh expression is localized in the proliferating population of the SVZ cells. Double labeling of shh and nestin immunostained elements revealed that a portion of the shh+ cells are also nestin+ in the SVZ (Fig 5D), suggesting that shh protein is expressed and produced in nestin+ type B cells in the SVZ. Shh expression was also detected in DCX+ neuroblasts (Fig 5E). Since smo and ptc proteins are also expressed in the SVZ NSCs niche, we examined whether shh producing cells also express the shh receptor and/or effector. In fact, shh immunoreactivity was also detected both in smo positive (Fig 5F) and ptc positive cells (Fig 5G), suggesting a possible autocrine mechanism for the regulation of the shh signaling pathway within the SVZ niche.

### Modulation of shh signaling pathway after cortical ischemic injury alters behavioral function

**Fig 6:**
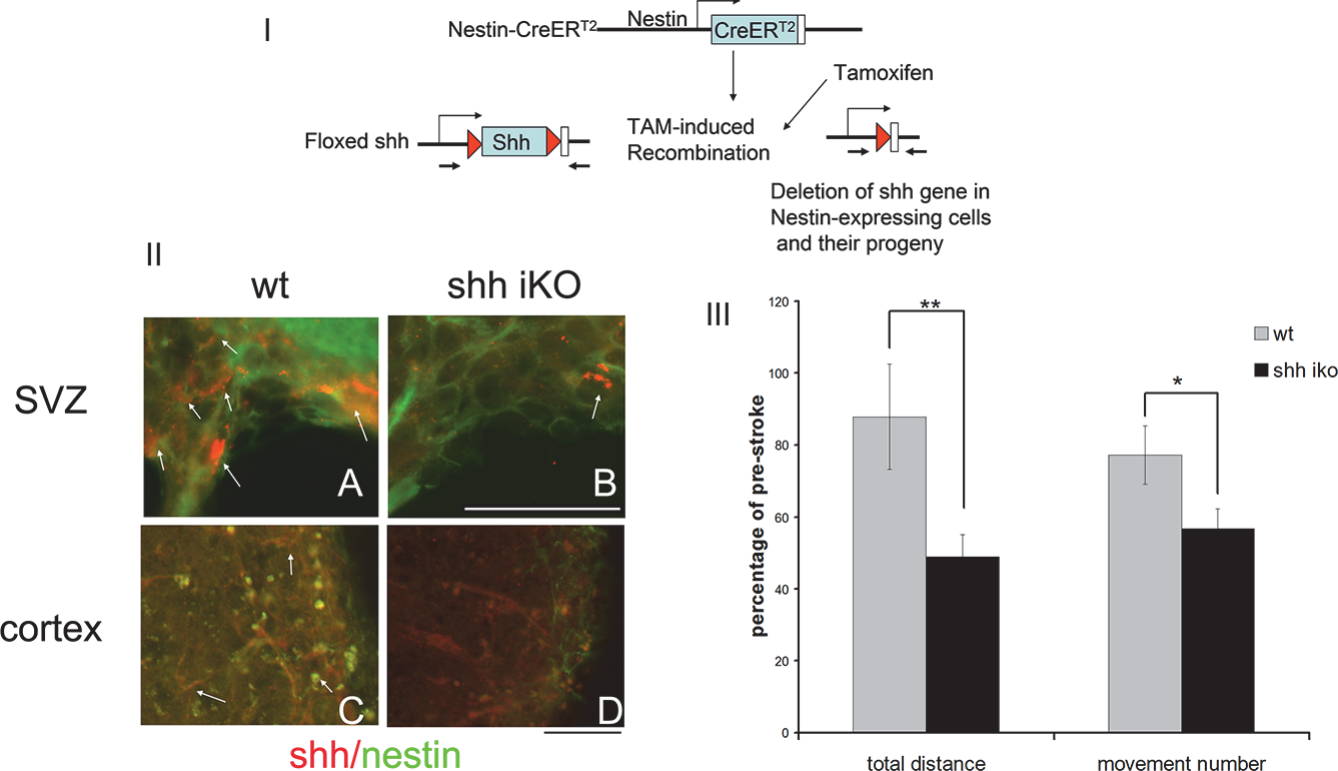
Nestin(+) cell-specific deletion of shh gene leads to more motor function deficits in shh iKO mice. I) strategy for nestin-expressing cell specific deletion of shh. II) in wt mice, shh expression is detected in many cells that are also nestin(+) in the SVZ (A) and in the ischemic site of the cortical stroke area (C). However, in shh iKO mice, only very few nestin positive cells express shh (panel B arrow). Similarly at the ischemic site in cortex (D), nestin (+) cells do not express shh while surrounding cells expressing shh are nestin negative. This confirms the deletion of the shh gene in both SVZ and cortical nestin-expressing cells near the ischemic site. III) Deletion of shh gene in nestin(+) cells in shh iKO mice leads to greater motor deficits in these ko mice compared to wt mice measured by total distance travelled and total movement number recorded in 24 hours at post-stroke day 7. Data normalized to pre-stroke value of each animal and presented as percentage of pre-stroke measurements. (** indicates p<0.01, * indicates p<0.05, n = 7–8, Student’s t-test). Scale bar = 50um.

To test whether modulation of the shh signaling pathway alters the behavioral outcome of cortical stroke, we utilized the same nestincreERT2 mouse line to conditionally delete shh gene in aged shh inducible knockout (iKO) mice. Since MCAo surgery was performed 3 days after the last administration of TAM (Fig 6I), we speculated that, with this treatment regimen, the shh gene is deleted both in SVZ NSCs as well as nestin-expressing cells activated in cortical regions near the ischemic site. Indeed, when stained with shh and nestin antibodies in post-stroke brain, we identified a number of cells in the SVZ in wt mice that are nestin+/shh+ (Fig 6II panel A, arrows) but only a few cells (Fig 6II panel B, arrow) in shh iKO animals to be shh positive, suggesting deletion of the shh gene in the SVZ niche. In addition, we found that many nestin+ cells near the ischemic core are shh positive (Fig 6II panel C, arrows) in wt mice. In contrast, nestin+ cells in TAM treated iKO mice have no or minimum shh expression (arrow head in Fig 6II panel D) while some surrounding cells that are nestin negative express shh protein (short arrows in Fig 6II D). Thus, under this treatment regimen, shh gene deletion occurs both in SVZ nestin positive and cortical nestin upregulated cells.

Locomotor function measured before MCAo surgery showed no difference in shh iKO and wt mice (Total distance traveled = 37196.8±13160.17cm for wt and 32132.1±10981.2cm for ko mice, p = 0.79, Student’s t-test; Total movement number = 4702±1436.5 for wt and 4385±988.2 for ko mice, p = 0.86, Student’s t-test). However, locomotor measurements carried out on post-stroke day7 (psd7) demonstrated that shh iKO mice showed significantly more severe motor deficits compared to wt littermates both in total distance travelled and total movement number measured in 24-hour period (total distance traveled = 87.9±14.6% of pre-stroke level for wt mice and 48.9±6.3% of pre-stroke level for shh iKO mice, p = 0.020, Student’s t-test; Total movement number = 67.7±6.8% of pre-stroke level in wt and 50.0±8.5% in shh iKO mice, p = 0.014, Student’s t-test, Fig 6III n = 7–8).

**Fig 7:**
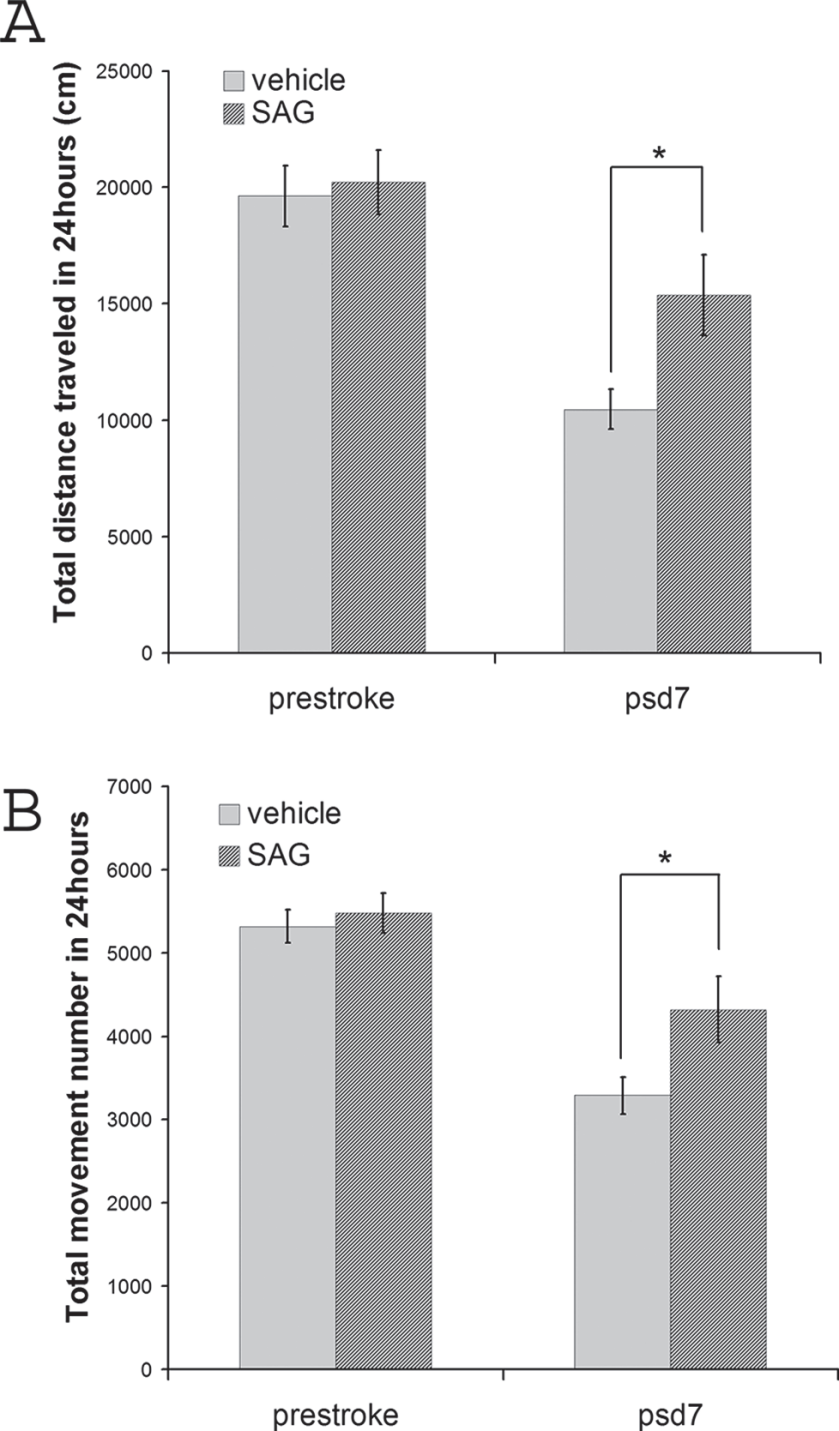
Shh pathway agonist treatment starting at post-stroke day (psd 3) improves motor function in stroke mice. Motor function was measured by total distance travelled (cm) and total movement number recorded in 24 hours. Measurement was carried out before MCAo surgery and on post-stroke day 7. (n = 15, * indicates p<0.05, Student’s t-test).

To further test whether activation of shh signaling alters the functional outcome of stroke, we utilized a smo agonist (SAG) that has been previously reported to activate the shh pathway and modify the proliferation of SVZ NSCs in vivo [22, 23]. 3 days after MCAo surgery, mice were subject to 1hr locomotion test and randomly placed into two equal groups. Pre-stroke and post-stroke day3 locomotion measurements both confirmed that there was no significant difference on basal level locomotion measurement and psd 3 locomotion deficits between the two groups before the initiation of vehicle or SAG treatment (total distance traveled = 19630.4±1302.3cm for vehicle group and 20217.0±1386.4 cm for SAG treatment group, p = 0.97; total movement number = 5316.9±200.7 for vehicle group and 5478.9±235.8 for SAG treatment group, p = 0.88, Student’s t-test, Fig 7A and 7B). After the behavioral test on psd 3, one of the groups received smo agonist treatment by gavage feeding (10mg/kg daily starting from psd3); the other group received vehicle (0.5% methyl cellulose, 0.2% Tween-80/dH2O). Similar behavioral measurements were carried out on psd 7 for 24 hours. Mice that received SAG treatment showed elevated locomotor activity compared to vehicle treated mice (total distance traveled = 10461.7±864.2cm for vehicle group and 15368.1±1741.3 cm for SAG treatment group, p = 0.02; total movement number = 3288.9±224.9 for vehicle group and 4317.3±396.8 for SAG treatment group, p = 0.03, Student’s t-test, Fig 7A and 7B, n = 15 for each group). Post-stroke treatment with a shh pathway agonist at psd3 after MCAo surgery thus supports that activation of the shh signaling pathway improves the behavioral outcome 7 days after ischemia onset.

## Discussion

We and others have previously reported that direct application in shh peptide resulted in significant improvement in neurological outcome after stroke [18–21]. The beneficial effects of shh pathway activation could be attributed to multiple aspects of the pleiotropic effects of shh, such as maintaining the integrity of the blood brain barrier [14] and/or, promoting the proliferation and survival of endogenous or exogenous neural progenitor cells in stroke animals [21, 34]. A recent study reported that shh signaling is also necessary and sufficient to elicit the stem cell response from locally activated astrocytes in injured brain[17]. The shh signaling pathway is upregulated in response to ischemia [35]; however, the precise source of shh and the cellular elements that respond to shh signaling have not been identified in focal ischemic models. Characterization and identification of the source of the shh signal and the responding cell types are the initial critical steps toward understanding the role of shh in regulating adult stem cells both in the SVZ niche as well as in activated astrocytes in situ that manifest stem cell potentials. In this study, we report upregulation of shh expression at both the cortical ischemic site as well as adjacent striatal regions. Cell types that upregulate shh expression include neurons, and activated astrocytes that also express nestin protein, as well as NSCs in the SVZ niche. In the SVZ niche, shh expression can be visualized in PCNA positive proliferating cells which include nestin positive self-renewable type B cells and DCX positive migrating neuroblasts. Our data also suggests that Shh might function in an autocrine fashion in the SVZ niche, affecting the cells in which it is produced [36] since both Shh+/smo+ cells and shh+/ptc+ cells are identified in the SVZ niche. Expression of shh protein in multiple cell types in the SVZ suggests that it might play an important role in multiple aspects of NSC regulation and maintenance.

Using a distal MCAo protocol, we generated focal ischemic injury that is restricted to the cortex in mouse brain. We found that GFAP positive astrocytes are activated both in the ischemic cortex as well as the striatal regions. Interestingly, many of the GFAP positive reactive astrocytes also express nestin, a marker for neural progenitor cells, suggesting that these reactive astrocytes are undergoing a process of acquiring progenitor cell properties. This is consistent with recent reports on a latent neurogenic program in astrocytes after brain injury [4, 17] Our data showed that abundant GFAP+/nestin+ cells both in the cortical and striatal areas upregulate shh expression, indicating that shh signaling may play an important role in the activation of astrocytes in response to cortical ischemic injury. It is noteworthy that, although focal cortical ischemia is able to stimulate the proliferation and possible neurogenesis of SVZ NSCs identified by YFP immunostaining as well as DCX positive neuroblasts at the SVZ niche, the migration of these neuroblasts towards the cortical ischemic region is minimal compared to the abundant DCX positive cells migrating into the corpus callosum (CC) and striatal areas in proximal MCAo-induced striatal stroke (Fig 1H–1L). Therefore, we speculate that local regenerative mechanisms might play a more important role in a focal cortical stroke. This data also might suggest that striatal stroke might be a better animal model to study the migration and integration of SVZ derived neurogenesis than cortical stroke.

Because aging is the most important risk factor for stroke, and aged patients and animals exhibit impaired stroke recovery [37, 38] to best mimic a clinically relevant scenario, we utilized aged mice [39, 40] (20–22 months) in which TAM administration enabled us to delete shh gene in nestin-expressing cells when TAM is present in vivo. Conditional deletion of shh in nestin-expressing cells resulted in more severe motor behavior deficits in shh iKO mice compared to their wt littermates at 7 days post stroke. The precise cellular component and molecular mechanisms that account for the more marked functional deficits need to be further investigated. We speculate that a combined contribution of SVZ progenitor cells and locally activated astrocytes that upregulate nestin expression may be involved. The TAM treatment regimen utilized in this study likely resulted in the deletion of shh genes both in the SVZ nestin-expressing cells and its progeny as well as nestin-upregulated astrocytes near the stroke site. It has reported that 1 week after the last TAM administration, recombination can still occur in 46–78% of the transplanted reporter cells [41]. In this study, when MCAo was carried out at 3 days after the last TAM injection, we observed loss of shh expression in nestin+ cells in the ischemic site in shh iKO mice but not in wt mice. Thus, shh signaling in nestin expressing cells after ischemic injury can alter the functional outcome of focal cortical ischemia. Given the distance of the SVZ to the cortical area affected by distal MCAo and the limited number of migrating DCX+ cells in the ischemic region, we speculate that the regional local nestin-expressing cells at the injury site that secret shh might contribute more substantially to the acute recovery outcome (7 days after distal MCAo) that we observed in this study. When TAM is given 14 days before the MCAo surgery to allow for the clearance of TAM from the body, recombinations of loxP flanked genes are restricted to the SVZ progenitor cells (unpublished observations), which allows for better targeted gene deletion in the SVZ progenitor cell population. Our lab is in the process of evaluating the effect of more restricted SVZ progenitor-specific shh gene deletion on longer term functional outcome in these animals. In addition, to examine the effect of local deletion of the shh gene in cortical nestin-upregulating cells after distal MCAo, it might be possible to inject tamoxifen into the cortical ischemic site after stroke to achieve restricted cortical but not SVZ nestin+ cell gene deletion. This option warrants testing in future studies.

Complementing the greater loss of motor function in shh inducible conditional knockout animals, treatment with a shh signaling agonist that activates the shh signaling pathway [22, 23] demonstrated efficacy in improving the behavioral outcome in stroke mice even when delivered 3 days after MCAo. Behavioral function was measured in automated activity chambers in this study, which is widely used to evaluate motor function in rodents [27, 42] and has demonstrated to positively correlate with ischemic injury in rats [43]. In this study, we observed changes consistent with the main function parameters in distal MCAo models in mice except for vertical activities, which showed the same trend but without statistical significance (see Table 1 for measurements and statistics for the first 8 primary motor parameters in vehicle and shh agonist treated mice). This could possibly be attributed to the large variation in vertical activities in mice and may suggest that horizontal movement parameters (total horizontal activity, distance travelled, movement numbers and movement times) might be more reliable variables to evaluate functional outcome in cortical stroke models. The precise molecular mechanism for the observed functional improvement in shh agonist treated stroke mice needs further investigation and the longer-term functional measurements also warrant follow up analysis. Since the shh signaling pathway has been implicated in maintaining the integrity of the blood brain barrier (BBB) [14, 15] and reactive glial responses after injury [16, 17], we speculate that the shh agonist might stimulate the repair and recovery of neurovasculature and mediate the proliferation and activation of astrocytes and microglial cells during stroke pathophysiology. Future studies that explore these and additional mechanisms are warranted. In summary, in this study, both the loss of function of shh in nestin-expressing cells and the activation of shh signaling pathways, taken together, demonstrate the critical role of the shh gene in the modulation of functional outcome and repair after focal cortical ischemic injury.

**Table 1:** Locomotor behavior parameters in vehicle and shh pathway agonist (SAG) treated mice before and 7 days after stroke.

* *	*Pre-stroke*						*Post-stroke*				
	*vehicle*		*SAG*			*vehicle*		*SAG*	
	*mean*	*s*.*e*.*m*.	*mean*	*s*.*e*.*m*.	* *p value*	*mean*	*s*.*e*.*m*.	*mean*	*s*.*e*.*m*.	* *p value*
HACTV	97746.1	3526.5	102555.9	4374.76	0.63	66280.2	4128.8	78303.9	4216.2	0.05
TOTDIST	19630.4	1302.3	20217	1386.4	0.97	10461.7	864.2	15368.1	1741.3	0.02
MOVNO	5316.9	200.7	5478.9	235.8	0.88	3288.9	224.9	4317.3	396.8	0.03
MOVTIME	2409.5	142.8	2493.2	151.2	0.96	1313.1	90.9	2019.4	259.3	0.019
RESTIME	83990.5	142.8	83906.8	151.3	0.96	85086.9	90.9	84380.6	259.3	0.019
VACTV	11201.4	592.2	11565.7	739.7	0.70	6962.9	719.4	8667.9	814.6	0.14
VMOVNO	2921.8	170.9	3208.1	154.2	0.22	1798.3	176.2	1932.9	100.7	0.52
VTIME	7576.0	300.2	7396.6	379.0	0.71	5090.2	490.4	5666.1	558.9	0.45

*Difference between vehicle and SAG treated mice within pre-stroke or post-stroke condition (Student’s t-test).

HACTV (horizontal activity) = total number of beam interruptions that occurred in the horizontal sensors

TOTDIST (total distance travelled) = the total distance (cm) travelled.

MOVNO (number of movements) = the number of separate horizontal movements executed.

MOVTIME (movement time) = the amount of time (second) in ambulation.

RESTIME (rest time) = the amount of time (second) in rest.

VACTV (vertical activity) = total number of beam interruptions that occurred in the vertical sensors.

VMOVNO (vertical number of movements) = the number of separate vertical movements executed.

VTIME (vertical movement time) = the amount of time (second) in vertical activity
